# Supplementary material for: Discovery and application of insertion-deletion (INDEL) polymorphisms for QTL mapping of early life-history traits in Atlantic salmon
Source: BMC Genomics. 2010 Mar 8;11:156. doi: 10.1186/1471-2164-11-156 (PMC2838853; doi:10.1186/1471-2164-11-156)
Supplement: Additional file 2 — Information on developed 76 locus single-run INDEL panel in Atlantic salmon. Information on fluorescence labeling, primer concentrations, PCR pooling and links to alignments, INDEL motifs and GENESCAN (Burge and Karlin 1997) predictions of genes/exons are available in html format. [file 1471-2164-11-156-S2.ZIP › Additionalfile2/table10023.html]

Contig Alignment

```
Base	A B C D E F G H I J K L M N O P Q   
1	G . G . . . . . G . . . . . . . .    
2	A . A . . . . . A . . . . . . . .    
3	T . T . . . . . T . . . . . . . .    
4	T . T . . . . . T . . . . . . . .    
5	T . T T . . . . T . . . . . . . .    
6	C . C C C C C C C . C . . . . . .    
7	A . A A A A A A A . A . . . . . .    
8	T . T T T T T T T . T . . . . . .    
9	A . A A A A A A A . A . . . . . .    
10	C . C C C C C C C . C . . . . . .    
11	G . G G G G G G G . G . . . . . .    
12	A . A A A A A A A . A . . . . . .    
13	T . T T T T T T T . T . . . . . .    
14	C . C C C C C C C . C . . . . . .    
15	A . A A A A A A A . A . . . . . .    
16	A . A A A A A A A . A . . . . . .    
17	T . T T T T T T T . T . . . . . .    
18	T . T T T T T T T . T . . . . . .    
19	T . T T T T T T T . T . . . . . .    
20	C . C C C C C C C . C . . . . . .    
21	T . T T T T T T T . T . . . . . .    
22	T . T T T T T T T . T . . . . . .    
23	T . T T T T T T T . T . . . . . .    
24	A . A A A A A A A . A . . . . . .    
25	T . T T T T T T T . T . . . . . .    
26	T . T T T T T T T . T . . . . . .    
27	A . A A A A A A A . A . . . . . .    
28	T . T T T T T T T . T . . . . . .    
29	A . A A A A A A A . A . . . . . .    
30	A . A A A A A A A . A . . . . . .    
31	T . T T T T T T T . T . . . . . .    
32	C . C C C C C C C . C . . . . . .    
33	C . C C C C C C C . C . . . . . .    
34	A . A A A A A A A . A . . . . . .    
35	T . T T T T T T T . T . . . . . .    
36	C . C C C C C C C . C . . . . . .    
37	A . A A A A A A A . A . . . . . .    
38	A . A A A A A A A . A . . . . . .    
39	C . C C C C C C C . C . . . . . .    
40	C . C C C C C C C . C . . . . . .    
41	G . G G G G G G G . G . . . . . .    
42	G . G G G G G G G . G . . . . . .    
43	A . A A A A A A A . A . . . . . .    
44	G . G G G G G G G . G . . . . . .    
45	G . G G G G G G G . G . . . . . .    
46	C . C C C C C C C . C . . . . . .    
47	C . C C C C C C C . C . . . . . .    
48	T . T T T T T T T . T . . . . . .    
49	G . G G G G G G G . G . . . . . .    
50	A . A A A A A A A . A . . . . . .    
51	C . C C C C C C C . C . . . . . .    
52	A . A A A A A A A . A . . . . . .    
53	A . A A A A A A A . A . . . . . .    
54	A . A A A A A A A . A . . . . . .    
55	G . G G G G G G G . G . . . . . .    
56	A A G G G G G G G . G . . . . . .    
57	A A A A A A A A A . A A . . . . .    
58	A A A A A A A A A . A A . . . . .    
59	A A A A A A A A A . A A . . . . .    
60	A A A A A A A A A . A A . . . . .    
61	A A A A A A A A A . A A . . . . .    
62	A A A A A A A A A . A A . . . . .    
63	A A A A A A A A A . A A . . . . .    
64	A A A A A A A A A . A A . . . . .    
65	C C C C C C C C C . C C . . . . .    
66	A A A A A A A A A . A A . . . . .    
67	G G G G G G G G G . G G . . . . .    
68	C C C C C C C C C . C C . . . . .    
69	T T T T T T T T T . T T . . . . .    
70	T T T T T T T T T . T T . . . . .    
71	T T T T T T T T T . T T . . . . .    
72	A A A A A A A A A . A A . . . . .    
73	A A A A A A A A A . A A . . . . .    
74	C C C C C C C C C . C C . . . . .    
75	T T T T T T T T T . T T . . . . .    
76	C C C C C C C C C . C C . . . . .    
77	A A A A A A A A A . A A . . . . .    
78	A A A A A A A A A . A A . . . . .    
79	C C C C C C C C C . C C . . . . .    
80	C C C C C C C C C . C C . . . . .    
81	C C C C C C C C C . C C . . . . .    
82	C C C C C C C C C . C C . . . . .    
83	T T T T T T T T T . T T . . . . .    
84	A A A A A A A A A . A A . . . . .    
85	C C C C C C C C C . C C . . . . .    
86	T T T T T T T T T . T T . . . . .    
87	T T T T T T T T T . T T . . . . .    
88	A A A A A A A A A . A A . . . . .    
89	G G G G G G G G G . G G . . . . .    
90	T T T T T T T T T . T T . . . . .    
91	T T T T T T T T T . T T . . . . .    
92	T T T T T T T T T . T T . . . . .    
93	C C C C C C C C C . C C . . . . .    
94	C C C C C C C C C . C C . . . . .    
95	C C C C C C C C C . C C . . . . .    
96	A A A A A A A A A . A A . . . . .    
97	G G G G G G G G G . G G . . . . .    
98	G G G G G G G G G . G G . . . . .    
99	C C C C C C C C C . C C . . . . .    
100	T T T T T T T T T . T T . . . . .    
101	T T T T T T T T T . T T . . . . .    
102	T T T T T T T T T . T T . . . . .    
103	C C C C C C C C C . C C . . . . .    
104	T T T T T T T T T . T T . . . . .    
105	C C C C C C C C C . C C . . . . .    
106	A A A A A A A A A . A A . . . . .    
107	C C C C C C C C C . C C . . . . .    
108	G G G G G G G G G . G G . . . . .    
109	A A A A A A A A A . A A . . . . .    
110	T T T T T T T T T . T T . . . . .    
111	T T T T T T T T T . T T . . . . .    
112	T T T T T T T T T . T T . . . . .    
113	C C C C C C C C C . C C . . . . .    
114	A A A A A A A A A . A A . . . . .    
115	T T T T T T T T T . T T . . . . .    
116	T T T T T T T T T . T T . . . . .    
117	T T T T T T T T T . T T . . . . .    
118	T T T T T T T T T . T T . . . . .    
119	G G G G G G G G G . G G . . . . .    
120	T T T T T T T T T . T T . . . . .    
121	T T T T T T T T T . T T . . . . .    
122	C C C C C C C C C . C C . . . . .    
123	C C C C C C C C C . C C . . . . .    
124	A A A A A A A A A . A A . . . . .    
125	A A A A A A A A A . A A . . . . .    
126	T T T T T T T T T . T T . . . . .    
127	A A A A A A A A A . A A . . . . .    
128	G G G G G G G G G G G G . . . . .    
129	C C C C C C C C C C C C . . . . .    
130	T T T T T T T T T T T T . . . . .    
131	G G G G G G G G G G G G . . . . .    
132	A A A A A A A A A A A A . . . . .    
133	G G G G G G G G G G G G . . . . .    
134	T T T T T T T T T T T T . . . . .    
135	G G G G G G G G G G G G . . . . .    
136	A A A A A A A A A A A A . . . . .    
137	T T T T T T T T T T T T . . . . .    
138	G G G G G G G G G G G G . . . . .    
139	A A A A A A A A A A A A . . . . .    
140	A A A A A A A A A A A A . . . . .    
141	C C C C C C C C C C C C . . . . .    
142	G G G G G G G G G G G G . . . . .    
143	A A A A A A A A A A A A . . . . .    
144	G G G G G G G G G G G G . . . . .    
145	G G G G G G G G G G G G . . . . .    
146	A A A A A A A A A A A A . . . . .    
147	T T T T T T T T T T T T . . . . .    
148	T T T T T T T T T T T T . . . . .    
149	G G G G G G G G G G G G . . . . .    
150	G G G G G G G G G G G G . . . . .    
151	A A A A A A A A A A A A . . . . .    
152	A A A A A A A A A A A A . . . . .    
153	G G G G G G G G G G G G . . . . .    
154	G G G G G G G G G G G G . . . . .    
155	A A A A A A A A A A A A . . . . .    
156	T T T T T T T T T T T T . . . . .    
157	G G G G G G G G G G G G . . . . .    
158	G G G G G G G G G G G G . . . . .    
159	C C C C C C C C C C C C . . . . .    
160	A A A A A A A A A A A A . . . . .    
161	T T T T T T T T T T T T . . . . .    
162	C C C C C C C C C C C C . . . . .    
163	G G G G G G G G G G G G . . . . .    
164	C C C C C C C C C C C C . . . . .    
165	A A A A A A A A A A A A . . . . .    
166	A A A A A A A A A A A A . . . . .    
167	G G G G G G G G G G G G . . . . .    
168	A A A A A A A A A A A A . . . . .    
169	C C C C C C C C C C C C . . . . .    
170	T T T T T T T T T T T T . . . . .    
171	A A A A A A A A A A A A . . . . .    
172	C C C C C C C C C C C C . . . . .    
173	G G G G G G G G C G G G . . . . .    
174	C C C C C C C C C C C C . . . . .    
175	A A A A A A A A A A A A . . . . .    
176	C C C C C C C C C C C C . . . . .    
177	C C C C C C C C C C C C . . . . .    
178	C C C C C C C C C C C C . . . . .    
179	C C C C C C C C C C C C . . . . .    
180	A A A A A A A A A A A A . . . . .    
181	A A A A A A A A A A A A . . . . .    
182	A A A A A A A A A A A A . . . . .    
183	A A A A A A A A A A A A . . . . .    
184	A A A A A A A A A A A A . . . . .    
185	G G G G G G G G G G G G . . . . .    
186	A A A A A A A A A A A A . . . . .    
187	A A A A A A A A A A A A . . . . .    
188	A A A A A A A A A A A A . . . . .    
189	A A A A A A A A A A A A . . . . .    
190	C C C C C C C C C C C C . . . . .    
191	A A A A A A A A A A A A . . . . .    
192	C C C C C C C C C C C C . . . . .    
193	A A A A A A A A A A A A . . . . .    
194	G G G G G G G G G G G G . . . . .    
195	C C C C C C C C C C C C . . . . .    
196	A A A A A A A A A A A A . . . . .    
197	C C C C C C C C C C C C . . . . .    
198	T T T T T T T T T T T T . . . . .    
199	G G G G G G G G G G G G . . . . .    
200	C C C C C C C C C C C C . . . . .    
201	A A A A A A A A A A A A . . . . .    
202	C C C C C C C C C C C C . . . . .    
203	T T T T T T T T T T T T . . . . .    
204	G G G G G G G G G G G G . . . . .    
205	A A A A A A A A A A A A . . . . .    
206	A A A A A A A A A A A A . . . . .    
207	A A A A A A A A A A A A . . . . .    
208	A A A A A A A A A A A A . . . . .    
209	C C C C C C C C C C C C . . . . .    
210	A A A A A A A A A A A A . . . . .    
211	C C C C C C C C C C C C . . . . .    
212	A A A A A A A A A A A A . . . . .    
213	G G G G G G G G G G G G . . . . .    
214	A A A A A A A A A A A A . . . . .    
215	A A A A A A A A A A A A . . . . .    
216	C C C C C C C C C C C C . . . . .    
217	T T T T T T T T T T T T . . . . .    
218	T T T T T T T T T T T T . . . . .    
219	A A A A A A A A A A A A . . . . .    
220	A A A A A A A A A A A A . . . . .    
221	A A A A A A A A A A A A . . . . .    
222	A A A A A A A A A A A A . . . . .    
223	C C C C C C C C C C C C . . . . .    
224	C C C C C C C C C C C C . . . . .    
225	A A A A A A A A A A A A . . . . .    
226	C C C C C C C C C C C C . . . . .    
227	A A A A A A A A A A A A . . . . .    
228	A A A A A A A A A A A A . . . . .    
229	T T T T T T T T T T T T . . . . .    
230	A A A A A A A A A A A A . . . . .    
231	T T T T T T T T T T T T . . . . .    
232	T T T T T T T T T T T T . . . . .    
233	G G G G G G G G G G G G . . . . .    
234	T T T T T T T T T T T T . . . . .    
235	C C C C C C C C C C C C . . . . .    
236	A A A A A A A A A A A A . . . . .    
237	A A A A A A A A A A A A . . . . .    
238	C C C C C C C C C C C C . . . . .    
239	A A A A A A A A A A A A . . . . .    
240	A A A A A A A A A A A A . . . . .    
241	T T T T T T T T T T T T . . . . .    
242	G G G G G G G G G G G G . . . . .    
243	G G G G G G G G G G G G . . . . .    
244	C C T C C C C C C C C C . . . . .    
245	G G G G G G G G G G G G . . . . .    
246	G G G G G G G G G G G G . . . . .    
247	G G G G G G G G G G G G . . . . .    
248	T T T T T T T T T T T T . . . . .    
249	T T T T T T T T T T T T . . . . .    
250	C C C C C C C C C C C C . . . . .    
251	A A A A A A A A A A A A . . . . .    
252	T T T T T T T T T T T T . . . . .    
253	G G G G G G G G G G G G . . . . .    
254	G G G G G G G G G G G G . . . . .    
255	T T T T T T T T T T T T . . . . .    
256	T T T T T T T T T T T T . . . . .    
257	T T T T T T T T T T T T . . . . .    
258	T T T T T T T T T T T T . . . . .    
259	T T T T T T T T T T T T . . . . .    
260	T T T T T T T T T T T T . . . . .    
261	T T T T T T T T T T - T . . . . .    
262	T T T T - - T T T T - - . . . . .    
263	G G G G G G G G G G G G . . . . .    
264	G G G G G G G G G G G G . . . . .    
265	T T T T T T T T T T T T . . . . .    
266	C C C C C C C C C C C C . . . . .    
267	A A A A A A A A A A A A . . . . .    
268	C C C C C C C C C C C C . . . . .    
269	A A C A A A A A A A A A . . . . .    
270	T T C T T T T T T T T T . . . . .    
271	T T T T T T T T T T T T . . . . .    
272	T T T T T T T T T T T T . . . . .    
273	A A A A A A A A A A A A . . . . .    
274	C C C C C C C C C C C C . . . . .    
275	A A A A A A A A A A A A . . . . .    
276	C C C C C C C C C C C C . . . . .    
277	A A A A A A A A A A A A . . . . .    
278	A A A A A A A A A A A A . . . . .    
279	T T T T T T T T T T T T . . . . .    
280	G G G G G G G G G G G G . . . . .    
281	G G G G G G G G G G G G . . . . .    
282	T T T T T T T T T T T T . . . . .    
283	C C C C C C C C C C C C . . . . .    
284	A A A A A A A A A A A A . . . . .    
285	A A A A A A A A A A A A . . . . .    
286	G G G G G G G G G G G G . . . . .    
287	T T T T T T T T T T T T . . . . .    
288	T T T T T T T T T T T T . . . . .    
289	G G G G G G G G G G G G . . . . .    
290	T T T T T T T T T T T T . . . . .    
291	C C C C C C C C C C C C . . . . .    
292	T T T T T T T T T T T T . . . . .    
293	T T T T T T T T T T T T . . . . .    
294	T T T T T T T T T T T T . . . . .    
295	T T T T T T T T T T T T . . . . .    
296	G G G G G G G G G G G G . . . . .    
297	A A A A A A A A A A A A . . . . .    
298	T T T T T T T T T T T T . . . . .    
299	A A A A A A A A A A A A . . . . .    
300	A A A A A A A A A A A A . . . . .    
301	T T T T T T T T T T T T . . . . .    
302	T T T T T T T T T T T T . . . . .    
303	G G G G G G G G G G G G . . . . .    
304	T T T T T T T T T T T T . . . . .    
305	T T T T T T T T T T T T . . . . .    
306	A A A A A A A A A A A A . . . . .    
307	C C C C C C C C C C C C . . . . .    
308	A A A A A A A A A A A A . . . . .    
309	C C C C C C C C C C C C . . . . .    
310	A A A A A A A A A A A A . . . . .    
311	A A A A A A A A A A A A . . . . .    
312	T T T T T T T T T T T T . . . . .    
313	T T T T T T T T T T T T . . . . .    
314	G G G G G G G G G G G G . . . . .    
315	T T T T T T T T T T T T . . . . .    
316	C C C C C C C C C C C C . . . . .    
317	A A A A A A A A A A A A . . . . .    
318	G G G G G G G G G G G A . . . . .    
319	C C C C C C C C C C C C . . . . .    
320	A A A A A A A A A A A A . . . . .    
321	T T T T T T T T T T T T . . . . .    
322	T T T T T T T T T T T T . . . . .    
323	T T T T T T T T T T T T . . . . .    
324	G G G G G G G G G G G G . . . . .    
325	A A A A A A A A A A A A . . . . .    
326	G G G G G G G G G G G G . . . . .    
327	G G G G G G G G G G G G . . . . .    
328	T T T T T T T T T T T T . . . . .    
329	T T T T T T T T T T T T . . . . .    
330	G G G G G G G G G G G G . . . . .    
331	A A A A A A A A A A A A . . . . .    
332	T T T T T T T T T T T T . . . . .    
333	A A A A A A A A A A A A . . . . .    
334	A A A A A A A A A A A A . . . . .    
335	C C C C C C C C C C C C . . . . .    
336	A A A A A A A A A A A A . . . . .    
337	G G G G G G G G G G G G . . . . .    
338	G G G G G G G G G G G G . . . . .    
339	T T T T T T T T T T T T . . . . .    
340	T T T T T T T T T T T T . . . . .    
341	C C C C C C C C C C C C . . . . .    
342	T T T T T T T T T T T T . . . . .    
343	A A A A A A A A A A A A . . . . .    
344	T T T T T T T T T T T T . . . . .    
345	A A A A A A A A A A A A . . . . .    
346	G G G G G G G G G G G G . . . . .    
347	T T T T T T T T T T T T . . . . .    
348	C C C C C C C C C C C C . . . . .    
349	T T T T T T T T T T T T . . . . .    
350	A A A A A A A A A A A A . . . . .    
351	G G G G G G G G G G G G . . . . .    
352	G G G G G G G G G G G G . . . . .    
353	C C C C C C C C C C C C . . . . .    
354	G G G G G G G G G G G G . . . . .    
355	T T T T T T T T T T T T . . . . .    
356	G G G G G G G G G G G G . . . . .    
357	C C C C C C C C C C C C . . . . .    
358	T T T T T T T T T T T T . . . . .    
359	G G G G G G G G G G G G . . . . .    
360	T T T T T T T T T T T T . . . . .    
361	C C C C C C C C C C C C . . . . .    
362	G G G G G G G G G G G G . . . . .    
363	T T T T T T T T T T T T . . . . .    
364	T T T T T T T T T T T T . . . . .    
365	C C C C C C C C C C C C . . . . .    
366	C C C C C C C C C C C C . . . . .    
367	A A A A A A A A A A A A . . . . .    
368	T T T T T T T T T T T T . . . . .    
369	C C C C C C C C C C C C . . . . .    
370	G G G G G G G G G G G G . . . . .    
371	G G G G G G G G G G G G . . . . .    
372	C C C C C C C C C C C C . . . . .    
373	A A A A A A A A A A A A . . . . .    
374	G G G G G G G G G G G G . . . . .    
375	A A A A A A A A A A A A . . . . .    
376	C C C C C C C C C C C C . . . . .    
377	A A A A A A A A A A A A . . . . .    
378	T T T T T T T T T T T T . . . . .    
379	G G G G G G G G G G G G . . . . .    
380	- - - - A A - - A A A A . . . . .  6	 indel  
381	- - - - A A - - A A A A . . . . .  6	 indel  
382	- - - - G G - - G G G G . . . . .  6	 indel  
383	A A A A A A A A A A A A . . . . .    
384	G G G G G G G G G G G G . . . . .    
385	G G G G G G G G G G G G . . . . .    
386	C C C C C C C C C C C C . . . . .    
387	T T T T T T T T T T T T . . . . .    
388	T T T T T T T T T T T T . . . . .    
389	T T T T T T T T T T T T . . . . .    
390	A A A A A A A A A A A A . . . . .    
391	G G G G G G G G G G G G . . . . .    
392	T T T T T T T T T T T T . . . . .    
393	A A A A A A A A A A A A . . . . .    
394	C C C C C C C C C C C C . . . . .    
395	A A A A A A A A A A A A . . . . .    
396	C C C C C C C C C C C C . . . . .    
397	A A A A A A A A A A A A . . . . .    
398	C C C C C C C C C C C C . . . . .    
399	A A A A A A A A A A A A . . . . .    
400	T T T T T T T T T T T T . . . . .    
401	G G G G G G G G G G G G . . . . .    
402	C C C C C C C C C C C C . . . . .    
403	A A A A A A A A A A A A . . . . .    
404	T T T T T T T T T T T T . . . . .    
405	T T T T T T T T T T T T . . . . .    
406	T T T T T T T T T T T T . . . . .    
407	G G G G G G G G G G G G . . . . .    
408	T T T T T T T T T T T T . . . . .    
409	C C C C C C C C C C C C . . . . .    
410	A A A A A A A A A A A A . . . . .    
411	T T T T T T T T T T T T . . . . .    
412	C C C C C C C C C C C C . . . . .    
413	G G G G G G G G G G G G . . . . .    
414	G G G G G G G G G G G G . . . . .    
415	G G G G G G G G G G G G . . . . .    
416	A A A A A A A A A A A A . . . . .    
417	T T T T T T T T T T T T . . . . .    
418	C C C C C C C C C C C C . . . . .    
419	A A A A A A A A A A A A . . . . .    
420	A A A A A A A A A A A A . . . . .    
421	A A A A A A A A A A A A . . . . .    
422	G G G G G G G G G G G G . . . . .    
423	T T T T T T T T T T T T . . . . .    
424	G G G G G G G G G G G G . . . . .    
425	A A A A A A A A A A A A . . . . .    
426	G G G G G G G G G G G G . . . . .    
427	G G G G G G G G G G G G . . . . .    
428	G G G G G G G G G G G G . . . . .    
429	T T T T T T T T T T T T . . . . .    
430	T T T T T T T T T T T T . . . . .    
431	G G G G G G G G G G G G . . . . .    
432	A A A A A A A A A A A A . . . . .    
433	C C C C C C C C C C C C . . . . .    
434	A A A A A A A A A A A A . . . . .    
435	T T T T T T T T T T T T . . . . .    
436	C C C C C C C C C C C C . . . . .    
437	A A A A A A A A A A A A . . . . .    
438	G G G G G G G G G G G G . . . . .    
439	T T T T T T T T T T T T . . . . .    
440	T T T T T T T T T T T T . . . . .    
441	T T T T T T T T T T T T . . . . .    
442	T T T T T T T T T T T T . . . . .    
443	A A A A A A A A A A A A . . . . .    
444	T T T T T T T T T T T T . . . . .    
445	A A A A A A A A A A A A . . . . .    
446	A A A A A A A A A A A A . . . . .    
447	T T T T T T T T T T T T . . . . .    
448	G G G G G G G G G G G G . . . . .    
449	C C C C C C C C C C C C . . . . .    
450	T T T T T T T T T T T T . . . . .    
451	C C C C C C C C C C C C . . . . .    
452	A A A A A A A A A A A A . . . . .    
453	A A A A A A A A A A A A . . . . .    
454	A A A A A A A A A A A A . . . . .    
455	C C C C C C C C C C C C . . . . .    
456	A A A A A A A A A A A A . . . . .    
457	G G G G G G G G G G G G . . . . .    
458	T T T T T T T T T T T T . . . . .    
459	C C C C C C C C C C C C . . . . .    
460	A A A A A A A A A A A A . . . . .    
461	A A A A A A A A A A A A . . . . .    
462	A A A A A A A A A A A A . . . . .    
463	A A A A A A A A A A A A . . . . .    
464	C C C C C C C C C C C C . . . . .    
465	T T T T T T T T T T T T . . . . .    
466	A A A A A A A A A A A A . . . . .    
467	A A A A A A A A A A A A . . . . .    
468	A A A A A A A A A A A A . . . . .    
469	A A A A A A A A A A A A . . . . .    
470	C C C C C C C C C C C C . . . . .    
471	T T T T T T T T T T T T . . . . .    
472	A A A A A A A A A A A A . . . . .    
473	T T T T T T T T T T T T . . . . .    
474	C C C C C C C C C C C C . . . . .    
475	A A A A A A A A A A A A . . . . .    
476	A A A A A A A A A A A A . . . . .    
477	C C C C C C C C C C C C . . . . .    
478	T T T T T T T T T T T T . . . . .    
479	A A A A A A A A A A A A . . . . .    
480	A A A A A A A A A A A A . . . . .    
481	C C C C C C C C C C C C . . . . .    
482	T T T T T T T T T T T T . T . . .    
483	A A A A A A A A A A A A . A . . .    
484	A A A A A A A A A A A A . A . . .    
485	T T T T T T T T T T T T . T . . .    
486	A A A A A A A A A A A A . A . . .    
487	T T T T T T T T T T T T . T . . .    
488	T T T T T T T T T T T T . T . . .    
489	T T T T T T T T T T T T . T . . .    
490	G G G G G G G G G G G G . G . . .    
491	T T T T T T T T T T T T . T . . .    
492	T T T T T T T T T T T T . T . . .    
493	A A A A A A A A A A A A . A . . .    
494	A A A A A A A A A A A A . A . . .    
495	T T T T T T T T T T T T . T . . .    
496	A A A A A A A A A A A A . A . . .    
497	A A A A A A A A A A A A . A . . .    
498	A A A A A A A A A A A A . A . . .    
499	A A A A A A A A A A A A . A . . .    
500	G G G G G G G G G G G G . G . . .    
501	C C C C C C C C C C C C . C . . .    
502	A A A A A A A A A A A A . A . . .    
503	A A A A A A A A A A A A . A . . .    
504	A A A A A A A A A A A A . A . . .    
505	T T T T T T T T T T T T . T . . .    
506	G G G G G G G G G G G G . G . . .    
507	G G G G G G G G G G G G . G . . .    
508	A A A A A A A A A A A A . A . . .    
509	A A A A A A A A A A A A . A . . .    
510	A A A A A A A A A A A A . A . . .    
511	C C C C C C C C C C C C . C . . .    
512	T T T T T T T T T T T T . T . . .    
513	G G G G G G G G G G G G . G . . .    
514	G G G G G G G G G G G G . G . . .    
515	G G G G G G G G G G G G . G . . .    
516	T T T T T T T T T T T T . T . . .    
517	T T T T T T T T T T T T . T . . .    
518	T T T T T T T T T T T T . T . . .    
519	A A A A A A A A A A A A . A . . .    
520	A A A A A A A A A A A A . A . . .    
521	T T T T T T T T T T T T . T . . .    
522	G G G G G G G G G G G G . G . . .    
523	A A A A A A A A A A A A . A . . .    
524	C C C C C C C C C C C C . C . . .    
525	A A A A A A A A A A A A . A . . .    
526	A A A A A A A A A A A A . A . . .    
527	T T T T T T T T T T T T . T . . .    
528	A A A A A A A A A A A A . A . . .    
529	T T T T T T T T T T T T . T . . .    
530	T T T T T T T T T T T T . T . . .    
531	C C C C C C C C C C C C . C . . .    
532	A A A A A A A A A A A A . A . . .    
533	G G G G G G G G G G G G . G . . .    
534	A A A A A A A A A A A A A A . . .    
535	A A A A A A A A A A A A A A . . .    
536	A A A A A A A A A A A A A A . . .    
537	G G G G G G G G G G G G G G . . .    
538	A A A A A A A A A A A A A A . . .    
539	T T T T T T T T T T T T T T . . .    
540	G G G G G G G G G G G G G G . . .    
541	A A A A A A A A A A A A A A . . .    
542	T T T T T T T T T T T T T T . . .    
543	T T T T T T T T T T T T T T . . .    
544	T T T T T T T T T T T T T T . . .    
545	C C C C C C C C C C C C C C . . .    
546	C C C C C C C C C C C C C C . . .    
547	A A A A A A A A A A A A A A . . .    
548	C C C C C C C C C C C C C C . . .    
549	A A A A A A A A A A A A A A . . .    
550	C C C C C C C C C C C C C C . . .    
551	C C C C C C C C C C C C C C . . .    
552	C C C C C C C C C C C C C C . . .    
553	A A A A A A A A A A A A A A . . .    
554	T T T T T T T T T T T T T T . . .    
555	T T T T T T T T T T T T T T . . .    
556	C C C C C C C C C C C C C C . . .    
557	T T T T T T T T T T T T T T . . .    
558	C C C C C C C C C C C C C C . . .    
559	A A A A A A A A A A A A A A . . .    
560	C C C C C C C C C C C C C C . . .    
561	T T T T T T T T T T T T T T . . .    
562	A A A A A A A A A A A A A A . . .    
563	C C A A A A A A A A A A A A . . .    
564	. . A A A A A A A A A A A A . . .    
565	. . A A A A A A A A A A A A . . .    
566	. . C C C C C C C C C C C C . . .    
567	. . T T T T T T T T T T T T . . .    
568	. . T T T T T T T T T T T T . . .    
569	. . A A A A A A A A A A A A . . .    
570	. . A A A A A A A A A A A A . . .    
571	. . A A A A A A A A A A A A . . .    
572	. . T T T T T T T T T T T T . . .    
573	. . A A A A A A A A A A A A . . .    
574	. . A A A A A A A A A A A A . . .    
575	. . T T T T T T T T T T T T . . .    
576	. . A A A A A A A A A A A A . . .    
577	. . T T T T T T T T T T T T . . .    
578	. . T T T T T T T T T T T T . . .    
579	. . T T T T T T T T T T T T . . .    
580	. . C C C C C C C C C C C C . . .    
581	. . A A A A A A A A A A A A . . .    
582	. . T T T T T T T T T T T T . . .    
583	. . . C C C C C C C C C C C . . .    
584	. . . C C C C C C C C C C C . . .    
585	. . . T T T T T T T T T T T . . .    
586	. . . A A A A A A A A A A A . . .    
587	. . . A A A A A A A A A A A . . .    
588	. . . A A A A A A A A A A A . . .    
589	. . . C C C C C C C C C C C . . .    
590	. . . A A A A A A A A A A A . . .    
591	. . . C C C C C C C C C C C . . .    
592	. . . A A A A A A A A A A A . . .    
593	. . . A A A A A A A A A A A . . .    
594	. . . G G G G G G G G G G G . . .    
595	. . . . C C C C C C C C C C . . .    
596	. . . . A A A A A A A A A A . . .    
597	. . . . T T T T T T T T T T . . .    
598	. . . . A A A A A A A A A A . . .    
599	. . . . C C C C C C C C C C . . .    
600	. . . . T T T T T T T T T T . . .    
601	. . . . T T T T T T T T T T . . .    
602	. . . . G G G G G G G G G G . . .    
603	. . . . C C C C C C C C C C . . .    
604	. . . . A A A A A A A A A A . . .    
605	. . . . T T T T T T T T T T . . .    
606	. . . . T T T T T T T T T T . . .    
607	. . . . A A A A A A A A A A . . .    
608	. . . . C C C C C C C C C C . . .    
609	. . . . T T T T T T T T T T . . .    
610	. . . . C C C C C C C C C C . . .    
611	. . . . A A A A A A A A A A . . .    
612	. . . . T T T T T T T T T T . . .    
613	. . . . C C C C C C C C C C . . .    
614	. . . . C C C C C C C C C C . . .    
615	. . . . C C C C C C C C C C . . .    
616	. . . . C C C C C C C C C C . . .    
617	. . . . C C C C C C C C C C . . .    
618	. . . . G G G G G G G G G G . . .    
619	. . . . G G G G G G G G G G . . .    
620	. . . . T T T T T T T T T T . . .    
621	. . . . C C C C C C C C C C . . .    
622	. . . . C C C C C C C C C C . . .    
623	. . . . C C A A A A A A A A . . .    
624	. . . . . . A A A A A A A A . . .    
625	. . . . . . T T T T T T T T . . .    
626	. . . . . . T T T T T T T T . . .    
627	. . . . . . T T T T T T T T . . .    
628	. . . . . . T T T T T T T T . . .    
629	. . . . . . T T T T T T T T . . .    
630	. . . . . . G G G G G G G G . . .    
631	. . . . . . A A A A A A A A . . .    
632	. . . . . . C C C C C C C C . . .    
633	. . . . . . C C C C C C C C . . .    
634	. . . . . . A A A A A A A A . . .    
635	. . . . . . A A A A A A A A . . .    
636	. . . . . . C C C C C C C C . . .    
637	. . . . . . C C C C C C C C . . .    
638	. . . . . . C C C C C C C C . . .    
639	. . . . . . C C C C C C C C . . .    
640	. . . . . . C C C C C C C C . . .    
641	. . . . . . C C C C C C C C . . .    
642	. . . . . . C C C C C C C C . . .    
643	. . . . . . C C C C C C C C . . .    
644	. . . . . . C C - - - - - - . . .    
645	. . . . . . C C - - - - - - . . .    
646	. . . . . . C C - - - - - - . . .    
647	. . . . . . C C - - - - - - . . .    
648	. . . . . . C C - - - - - - . . .    
649	. . . . . . C C - - - - - - . . .    
650	. . . . . . A A A A A A A A . . .    
651	. . . . . . G G G G G G G G . . .    
652	. . . . . . C C C C C C C C . . .    
653	. . . . . . G G G G G G G G . . .    
654	. . . . . . C C C C C C C C . . .    
655	. . . . . . A A A A A A A A . . .    
656	. . . . . . C C C C C C C C . . .    
657	. . . . . . T T T T T T T T . . .    
658	. . . . . . C C C C C C C C . . .    
659	. . . . . . T T T T T T T T . . .    
660	. . . . . . C C C C C C C C . . .    
661	. . . . . . G G G G G G G G . . .    
662	. . . . . . C C C C C C C C . . .    
663	. . . . . . A A A A A A A A . . .    
664	. . . . . . C C C C C C C C . . .    
665	. . . . . . G G G G G G G G . . .    
666	. . . . . . C C C C C C C C . . .    
667	. . . . . . A A A A A A A A . . .    
668	. . . . . . A A A A A A A A . . .    
669	. . . . . . C C C C C C C C . . .    
670	. . . . . . T T T T T T T T . . .    
671	. . . . . . G G G G G G G G . . .    
672	. . . . . . C C C C C C C C . . .    
673	. . . . . . G G G G G G G G . . .    
674	. . . . . . G G G G G G G G . . .    
675	. . . . . . T T T T T T T T . . .    
676	. . . . . . T T T T T T T T . . .    
677	. . . . . . G G G G G G G G . . .    
678	. . . . . . T T T T T T T T . . .    
679	. . . . . . T T T T T T T T . . .    
680	. . . . . . T T T T T T T T . . .    
681	. . . . . . T T T T T T T T . . .    
682	. . . . . . A A A A A A A A . . .    
683	. . . . . . A A A A A A A A . . .    
684	. . . . . . C . C C C C C C . . .    
685	. . . . . . . . A A A A A A . . .    
686	. . . . . . . . T T T T T T . . .    
687	. . . . . . . . A A A A A A . . .    
688	. . . . . . . . C C C C C C . . .    
689	. . . . . . . . A A A A A A . . .    
690	. . . . . . . . C C C T T C . . .    
691	. . . . . . . . A A A A A A . . .    
692	. . . . . . . . G G G G G G . . .    
693	. . . . . . . . C C C C C C . . .    
694	. . . . . . . . T T T T T T . . .    
695	. . . . . . . . G G G G G G . . .    
696	. . . . . . . . T T T T T T . . .    
697	. . . . . . . . A A A A A A . . .    
698	. . . . . . . . A A A A A A . . .    
699	. . . . . . . . C C C C C C . . .    
700	. . . . . . . . A A A A A A . . .    
701	. . . . . . . . C C C C C C . . .    
702	. . . . . . . . T T T T T T . . .    
703	. . . . . . . . G G G G G G . . .    
704	. . . . . . . . A A A A A A . . .    
705	. . . . . . . . C C C C C C . . .    
706	. . . . . . . . A A A A A A . . .    
707	. . . . . . . . A A A A A A . . .    
708	. . . . . . . . A A A A A A . . .    
709	. . . . . . . . G G G G G G . . .    
710	. . . . . . . . G G G G G G . . .    
711	. . . . . . . . C C C C C C . . .    
712	. . . . . . . . A A A A A A . . .    
713	. . . . . . . . T T T T T T . . .    
714	. . . . . . . . T T T T T T . . .    
715	. . . . . . . . A A A A A A . . .    
716	. . . . . . . . G G G G G G . . .    
717	. . . . . . . . C C C C C C . . .    
718	. . . . . . . . A A A A A A . . .    
719	. . . . . . . . T T T T T T . . .    
720	. . . . . . . . C C C C C C . . .    
721	. . . . . . . . T T T T T T . . .    
722	. . . . . . . . G G G G G G . . .    
723	. . . . . . . . T T T T T T . . .    
724	. . . . . . . . C C C C C C . . .    
725	. . . . . . . . C C C C C C . . .    
726	. . . . . . . . A A A A A A . . .    
727	. . . . . . . . T T T T T T . . .    
728	. . . . . . . . G G G G G G . . .    
729	. . . . . . . . C C C C C C . . .    
730	. . . . . . . . G G G G G G . . .    
731	. . . . . . . . C C C C C C . . .    
732	. . . . . . . . C C C C C C . . .    
733	. . . . . . . . C C C C C C . . .    
734	. . . . . . . . T T T T T T . . .    
735	. . . . . . . . T T T T T T . . .    
736	. . . . . . . . G G G G G G . . .    
737	. . . . . . . . C C C C C C . . .    
738	. . . . . . . . T T T T T T . . .    
739	. . . . . . . . C C C C C C . . .    
740	. . . . . . . . T T T T T T . . .    
741	. . . . . . . . C C C C C C . . .    
742	. . . . . . . . C C C C C C . . .    
743	. . . . . . . . G G A A A A . . .    
744	. . . . . . . . . . C C C C . . .    
745	. . . . . . . . . . G G G G . . .    
746	. . . . . . . . . . T T T T . . .    
747	. . . . . . . . . . T T T T . . .    
748	. . . . . . . . . . G G G G . . .    
749	. . . . . . . . . . G G G G . . .    
750	. . . . . . . . . . G G G G . . .    
751	. . . . . . . . . . T T T T . . .    
752	. . . . . . . . . . C C C C . . .    
753	. . . . . . . . . . A A A A . . .    
754	. . . . . . . . . . T T T T . . .    
755	. . . . . . . . . . T T T T . . .    
756	. . . . . . . . . . A A A A . . .    
757	. . . . . . . . . . A A A A . . .    
758	. . . . . . . . . . G G G G . . .    
759	. . . . . . . . . . T T T T . . .    
760	. . . . . . . . . . T T T T . . .    
761	. . . . . . . . . . A A A A . . .    
762	. . . . . . . . . . C C C C . . .    
763	. . . . . . . . . . T T T T . . .    
764	. . . . . . . . . . C C C C . . .    
765	. . . . . . . . . . C C C C . . .    
766	. . . . . . . . . . C C C C . . .    
767	. . . . . . . . . . C C C C . . .    
768	. . . . . . . . . . C C C C . . .    
769	. . . . . . . . . . A A A A . . .    
770	. . . . . . . . . . A A A A . . .    
771	. . . . . . . . . . C C C C . . .    
772	. . . . . . . . . . C C C C . . .    
773	. . . . . . . . . . A A A A . . .    
774	. . . . . . . . . . C C C C . . .    
775	. . . . . . . . . . A A A A . . .    
776	. . . . . . . . . . A A A A . . .    
777	. . . . . . . . . . C C C C . . .    
778	. . . . . . . . . . C C C C . . .    
779	. . . . . . . . . . G G G G . . .    
780	. . . . . . . . . . C C C C . . .    
781	. . . . . . . . . . T T T T . . .    
782	. . . . . . . . . . A A A A . . .    
783	. . . . . . . . . . C C C C . . .    
784	. . . . . . . . . . T T T T . . .    
785	. . . . . . . . . . C C C C . . .    
786	. . . . . . . . . . T T T T . . .    
787	. . . . . . . . . . T T T T . . .    
788	. . . . . . . . . . C C C C . . .    
789	. . . . . . . . . . A A A A . . .    
790	. . . . . . . . . . G G G G . . .    
791	. . . . . . . . . . G G G G . . .    
792	. . . . . . . . . . C C C C . . .    
793	. . . . . . . . . . A . A A . . .    
794	. . . . . . . . . . G . G G . . .    
795	. . . . . . . . . . C . C C . . .    
796	. . . . . . . . . . T . T T . . .    
797	. . . . . . . . . . G . G G . . .    
798	. . . . . . . . . . T . T T . . .    
799	. . . . . . . . . . A . A A . . .    
800	. . . . . . . . . . G . G G . . .    
801	. . . . . . . . . . T . T T . . .    
802	. . . . . . . . . . C . C C . . .    
803	. . . . . . . . . . C . C C . . .    
804	. . . . . . . . . . A . A A . . .    
805	. . . . . . . . . . A . A A . . .    
806	. . . . . . . . . . T . T T . . .    
807	. . . . . . . . . . . . G G . . .    
808	. . . . . . . . . . . . T T . . .    
809	. . . . . . . . . . . . C C . . .    
810	. . . . . . . . . . . . T T . . .    
811	. . . . . . . . . . . . A A . . .    
812	. . . . . . . . . . . . C C . . .    
813	. . . . . . . . . . . . A A . . .    
814	. . . . . . . . . . . . C C . . .    
815	. . . . . . . . . . . . A A . . .    
816	. . . . . . . . . . . . T T . . .    
817	. . . . . . . . . . . . G G . . .    
818	. . . . . . . . . . . . T T . . .    
819	. . . . . . . . . . . . A A . . .    
820	. . . . . . . . . . . . T T . . .    
821	. . . . . . . . . . . . C C . . .    
822	. . . . . . . . . . . . G G . . .    
823	. . . . . . . . . . . . T T . . .    
824	. . . . . . . . . . . . G G . . .    
825	. . . . . . . . . . . . C C . . .    
826	. . . . . . . . . . . . A A . . .    
827	. . . . . . . . . . . . G G . . .    
828	. . . . . . . . . . . . A A . . .    
829	. . . . . . . . . . . . A A . . .    
830	. . . . . . . . . . . . T T . . .    
831	. . . . . . . . . . . . C C . . .    
832	. . . . . . . . . . . . T T . . .    
833	. . . . . . . . . . . . A A . . .    
834	. . . . . . . . . . . . A A . . .    
835	. . . . . . . . . . . . T T . . .    
836	. . . . . . . . . . . . G G . . .    
837	. . . . . . . . . . . . C C . . .    
838	. . . . . . . . . . . . A A . . .    
839	. . . . . . . . . . . . T T . . .    
840	. . . . . . . . . . . . G G . . .    
841	. . . . . . . . . . . . T T . . .    
842	. . . . . . . . . . . . A A . . .    
843	. . . . . . . . . . . . C C . . .    
844	. . . . . . . . . . . . A A . . .    
845	. . . . . . . . . . . . T T . . .    
846	. . . . . . . . . . . . T T . . .    
847	. . . . . . . . . . . . A A . . A    
848	. . . . . . . . . . . . T T . . T    
849	. . . . . . . . . . . . A A . . A    
850	. . . . . . . . . . . . C C . . C    
851	. . . . . . . . . . . . A A . . A    
852	. . . . . . . . . . . . C C . . C    
853	. . . . . . . . . . . . A A . . A    
854	. . . . . . . . . . . . A A . . A    
855	. . . . . . . . . . . . G G . . G    
856	. . . . . . . . . . . . C C . . C    
857	. . . . . . . . . . . . A A . . A    
858	. . . . . . . . . . . . C C . . C    
859	. . . . . . . . . . . . T T . . T    
860	. . . . . . . . . . . . A A . . A    
861	. . . . . . . . . . . . T T . . T    
862	. . . . . . . . . . . . A A . . A    
863	. . . . . . . . . . . . G G . . G    
864	. . . . . . . . . . . . C C . C C    
865	. . . . . . . . . . . . C C . C C    
866	. . . . . . . . . . . . T T . T T    
867	. . . . . . . . . . . . G G . G G    
868	. . . . . . . . . . . . T T . T T    
869	. . . . . . . . . . . . G G . G G    
870	. . . . . . . . . . . . A A . A A    
871	. . . . . . . . . . . . G G . G G    
872	. . . . . . . . . . . . C C . C C    
873	. . . . . . . . . . . . G G . G G    
874	. . . . . . . . . . . . A A . A A    
875	. . . . . . . . . . . . G G . G G    
876	. . . . . . . . . . . . T T . T T    
877	. . . . . . . . . . . . G G . G G    
878	. . . . . . . . . . . . C C . C C    
879	. . . . . . . . . . . . A A . A A    
880	. . . . . . . . . . . . T T . T T    
881	. . . . . . . . . . . . T T . T T    
882	. . . . . . . . . . . . C C . C C    
883	. . . . . . . . . . . . T T . T T    
884	. . . . . . . . . . . . C C . C C    
885	. . . . . . . . . . . . A A . A A    
886	. . . . . . . . . . . . A A . A A    
887	. . . . . . . . . . . . A A . A A    
888	. . . . . . . . . . . . T T . T T    
889	. . . . . . . . . . . . G G . G G    
890	. . . . . . . . . . . . T T . T T    
891	. . . . . . . . . . . . G G . G G    
892	. . . . . . . . . . . . T T . T T    
893	. . . . . . . . . . . . G G . G G    
894	. . . . . . . . . . . . T T . T T    
895	. . . . . . . . . . . . A A . A A    
896	. . . . . . . . . . . . C C . C C    
897	. . . . . . . . . . . . A A . A A    
898	. . . . . . . . . . . . A A . A A    
899	. . . . . . . . . . . . T T . T T    
900	. . . . . . . . . . . . A A . A A    
901	. . . . . . . . . . . . T T . T T    
902	. . . . . . . . . . . . A A . A A    
903	. . . . . . . . . . . . T T . T T    
904	. . . . . . . . . . . . G G . G G    
905	. . . . . . . . . . . . T T . T T    
906	. . . . . . . . . . . . G G . G G    
907	. . . . . . . . . . . . A A . A A    
908	. . . . . . . . . . . . G G . G G    
909	. . . . . . . . . . . . G G . G G    
910	. . . . . . . . . . . . G G . G G    
911	. . . . . . . . . . . . G G . G G    
912	. . . . . . . . . . . . C C . C C    
913	. . . . . . . . . . . . G G . G G    
914	. . . . . . . . . . . . G G . G G    
915	. . . . . . . . . . . . A A . A A    
916	. . . . . . . . . . . . C C . C C    
917	. . . . . . . . . . . . A A . A A    
918	. . . . . . . . . . . . T T . T T    
919	. . . . . . . . . . . . A A . A A    
920	. . . . . . . . . . . . A A . A A    
921	. . . . . . . . . . . . T T . T T    
922	. . . . . . . . . . . . T T . T T    
923	. . . . . . . . . . . . A A . A A    
924	. . . . . . . . . . . . C C . C C    
925	. . . . . . . . . . . . A A . A A    
926	. . . . . . . . . . . . G G . G G    
927	. . . . . . . . . . . . G G . G G    
928	. . . . . . . . . . . . C C . C C    
929	. . . . . . . . . . . . T T . T T    
930	. . . . . . . . . . . . T T . T T    
931	. . . . . . . . . . . . G G . G G    
932	. . . . . . . . . . . . T T . T T    
933	. . . . . . . . . . . . C C . C C    
934	. . . . . . . . . . . . T T . T T    
935	. . . . . . . . . . . . A A . A A    
936	. . . . . . . . . . . . G G . G G    
937	. . . . . . . . . . . . C C . C C    
938	. . . . . . . . . . . . T T . T T    
939	. . . . . . . . . . . . T T . T T    
940	. . . . . . . . . . . . T T . T T    
941	. . . . . . . . . . . . A A . A A    
942	. . . . . . . . . . . . C C . C C    
943	. . . . . . . . . . . . A A . A A    
944	. . . . . . . . . . . . C C . C C    
945	. . . . . . . . . . . . A A . A A    
946	. . . . . . . . . . . . A A . A A    
947	. . . . . . . . . . . . T T . T T    
948	. . . . . . . . . . . . T T . T T    
949	. . . . . . . . . . . . C C . C C    
950	. . . . . . . . . . . . T T . T T    
951	. . . . . . . . . . . . T T . T T    
952	. . . . . . . . . . . . G G . G G    
953	. . . . . . . . . . . . C C . C C    
954	. . . . . . . . . . . . T T . T T    
955	. . . . . . . . . . . . G G . G G    
956	. . . . . . . . . . . . G G . G G    
957	. . . . . . . . . . . . T T . T T    
958	. . . . . . . . . . . . G G . G G    
959	. . . . . . . . . . . . G G . G G    
960	. . . . . . . . . . . . G G . G G    
961	. . . . . . . . . . . . A A . A A    
962	. . . . . . . . . . . . A A . A A    
963	. . . . . . . . . . . . G G . G G    
964	. . . . . . . . . . . . C C . C C    
965	. . . . . . . . . . . . T T . T T    
966	. . . . . . . . . . . . G G . G G    
967	. . . . . . . . . . . . A A . A A    
968	. . . . . . . . . . . . T T . T T    
969	. . . . . . . . . . . . A A . A A    
970	. . . . . . . . . . . . C C . C C    
971	. . . . . . . . . . . . A A . A A    
972	. . . . . . . . . . . . C C . C C    
973	. . . . . . . . . . . . A A . A A    
974	. . . . . . . . . . . . G G . G G    
975	. . . . . . . . . . . . G G . G G    
976	. . . . . . . . . . . . T T . T T    
977	. . . . . . . . . . . . A A . A A    
978	. . . . . . . . . . . . G G . G G    
979	. . . . . . . . . . . . T T . T T    
980	. . . . . . . . . . . . T T . T T    
981	. . . . . . . . . . . . G G . G G    
982	. . . . . . . . . . . . C C . C C    
983	. . . . . . . . . . . . C C . C C    
984	. . . . . . . . . . . . T T . T T    
985	. . . . . . . . . . . . A A . A A    
986	. . . . . . . . . . . . T T . T T    
987	. . . . . . . . . . . . A A . A A    
988	. . . . . . . . . . . . A A . A A    
989	. . . . . . . . . . . . G G . G G    
990	. . . . . . . . . . . . G G . G G    
991	. . . . . . . . . . . . A A . A A    
992	. . . . . . . . . . . . G G . G G    
993	. . . . . . . . . . . . A A . A A    
994	. . . . . . . . . . . . G G . G G    
995	. . . . . . . . . . . . G G . G G    
996	. . . . . . . . . . . . A A . A A    
997	. . . . . . . . . . . . G G . G G    
998	. . . . . . . . . . . . T T . T T    
999	. . . . . . . . . . . . G G . G G    
1000	. . . . . . . . . . . . T T . T T    
1001	. . . . . . . . . . . . A A . A A    
1002	. . . . . . . . . . . . G G . G G    
1003	. . . . . . . . . . . . G G . G G    
1004	. . . . . . . . . . . . G G . G G    
1005	. . . . . . . . . . . . G G . G G    
1006	. . . . . . . . . . . . A A . A A    
1007	. . . . . . . . . . . . C C . C C    
1008	. . . . . . . . . . . . A A . A A    
1009	. . . . . . . . . . . . C C . C C    
1010	. . . . . . . . . . . . T T . T T    
1011	. . . . . . . . . . . . G G . G G    
1012	. . . . . . . . . . . . G G . G G    
1013	. . . . . . . . . . . . G G . G G    
1014	. . . . . . . . . . . . G G . G G    
1015	. . . . . . . . . . . . A A . A A    
1016	. . . . . . . . . . . . T T . T T    
1017	. . . . . . . . . . . . T T . T T    
1018	. . . . . . . . . . . . G G . G G    
1019	. . . . . . . . . . . . G G . G G    
1020	. . . . . . . . . . . . G G . G G    
1021	. . . . . . . . . . . . A A . A A    
1022	. . . . . . . . . . . . G G . G G    
1023	. . . . . . . . . . . . A A . A A    
1024	. . . . . . . . . . . . G G . G G    
1025	. . . . . . . . . . . . A A . A A    
1026	. . . . . . . . . . . . C C . C C    
1027	. . . . . . . . . . . . G G . G G    
1028	. . . . . . . . . . . . T T . T T    
1029	. . . . . . . . . . . . T T . T T    
1030	. . . . . . . . . . . . G G . G G    
1031	. . . . . . . . . . . . G G . G G    
1032	. . . . . . . . . . . . G G . G G    
1033	. . . . . . . . . . . . T T . T T    
1034	. . . . . . . . . . . . G G . G G    
1035	. . . . . . . . . . . . C C . C C    
1036	. . . . . . . . . . . . A A . A A    
1037	. . . . . . . . . . . . C C . C C    
1038	. . . . . . . . . . . . A A . A A    
1039	. . . . . . . . . . . . A A . A A    
1040	. . . . . . . . . . . . G G . G G    
1041	. . . . . . . . . . . . G G . G G    
1042	. . . . . . . . . . . . C C . C C    
1043	. . . . . . . . . . . . A A . A A    
1044	. . . . . . . . . . . . G G . G G    
1045	. . . . . . . . . . . . A A . A A    
1046	. . . . . . . . . . . . A A . A A    
1047	. . . . . . . . . . . . A A . A A    
1048	. . . . . . . . . . . . G G . G G    
1049	. . . . . . . . . . . . G G . G G    
1050	. . . . . . . . . . . . A A . A A    
1051	. . . . . . . . . . . . G G . G G    
1052	. . . . . . . . . . . . G G . G G    
1053	. . . . . . . . . . . . G G . G G    
1054	. . . . . . . . . . . . A A . A A    
1055	. . . . . . . . . . . . C C . C C    
1056	. . . . . . . . . . . . G G . G G    
1057	. . . . . . . . . . . . G G . G G    
1058	. . . . . . . . . . . . G G . G G    
1059	. . . . . . . . . . . . C C . C C    
1060	. . . . . . . . . . . . G G . G G    
1061	. . . . . . . . . . . . A A . A A    
1062	. . . . . . . . . . . . C C . C C    
1063	. . . . . . . . . . . . T T . T T    
1064	. . . . . . . . . . . . C C . C C    
1065	. . . . . . . . . . . . A A . A A    
1066	. . . . . . . . . . . . G G . G G    
1067	. . . . . . . . . . . . G G . G G    
1068	. . . . . . . . . . . . A A . A A    
1069	. . . . . . . . . . . . G G . G G    
1070	. . . . . . . . . . . . T T . T T    
1071	. . . . . . . . . . . . T T . T T    
1072	. . . . . . . . . . . . A A . A A    
1073	. . . . . . . . . . . . T T . T T    
1074	. . . . . . . . . . . . C C . C C    
1075	. . . . . . . . . . . . A A . A A    
1076	. . . . . . . . . . . . G G . G G    
1077	. . . . . . . . . . . . A A . A A    
1078	. . . . . . . . . . . . G G . G G    
1079	. . . . . . . . . . . . G G . G G    
1080	. . . . . . . . . . . . G G . G G    
1081	. . . . . . . . . . . . C C . C C    
1082	. . . . . . . . . . . . A A . A A    
1083	. . . . . . . . . . . . C C . C C    
1084	. . . . . . . . . . . . A A . A A    
1085	. . . . . . . . . . . . T T . T T    
1086	. . . . . . . . . . . . G G . G G    
1087	. . . . . . . . . . . . C C . C C    
1088	. . . . . . . . . . . . T T . T T    
1089	. . . . . . . . . . . . C C . C C    
1090	. . . . . . . . . . . . C C . C C    
1091	. . . . . . . . . . . . T T . T T    
1092	. . . . . . . . . . . . C C . C C    
1093	. . . . . . . . . . . . G G . G G    
1094	. . . . . . . . . . . . A A . A A    
1095	. . . . . . . . . . . . A A . A A    
1096	. . . . . . . . . . . . G G . G G    
1097	. . . . . . . . . . . . C C . C C    
1098	. . . . . . . . . . . . C C . C C    
1099	. . . . . . . . . . . . C C . C C    
1100	. . . . . . . . . . . . T T . T T    
1101	. . . . . . . . . . . . C C . C C    
1102	. . . . . . . . . . . . G G . G G    
1103	. . . . . . . . . . . . C C . C C    
1104	. . . . . . . . . . . . T T . T T    
1105	. . . . . . . . . . . . C C . C C    
1106	. . . . . . . . . . . . T T . T T    
1107	. . . . . . . . . . . . C C . C C    
1108	. . . . . . . . . . . . C C . C C    
1109	. . . . . . . . . . . . C C . C C    
1110	. . . . . . . . . . . . T T . T T    
1111	. . . . . . . . . . . . T T . T T    
1112	. . . . . . . . . . . . A A . A A    
1113	. . . . . . . . . . . . C C . C C    
1114	. . . . . . . . . . . . T T . T T    
1115	. . . . . . . . . . . . C A . A A    
1116	. . . . . . . . . . . . . G . G G    
1117	. . . . . . . . . . . . . C . C C    
1118	. . . . . . . . . . . . . G . G G    
1119	. . . . . . . . . . . . . C . C C    
1120	. . . . . . . . . . . . . C . C C    
1121	. . . . . . . . . . . . . C . C C    
1122	. . . . . . . . . . . . . T . T T    
1123	. . . . . . . . . . . . . T . T T    
1124	. . . . . . . . . . . . . T . T T    
1125	. . . . . . . . . . . . . C . C C    
1126	. . . . . . . . . . . . . G . G G    
1127	. . . . . . . . . . . . . A . A A    
1128	. . . . . . . . . . . . . G . G G    
1129	. . . . . . . . . . . . . G . G G    
1130	. . . . . . . . . . . . . A . A A    
1131	. . . . . . . . . . . . . T . T T    
1132	. . . . . . . . . . . . . G . G G    
1133	. . . . . . . . . . . . . A . A A    
1134	. . . . . . . . . . . . . C . C C    
1135	. . . . . . . . . . . . . G . G G    
1136	. . . . . . . . . . . . . C . C C    
1137	. . . . . . . . . . . . . G . G G    
1138	. . . . . . . . . . . . . G . G G    
1139	. . . . . . . . . . . . . C . C C    
1140	. . . . . . . . . . . . . C . C C    
1141	. . . . . . . . . . . . . T . T T    
1142	. . . . . . . . . . . . . T . T T    
1143	. . . . . . . . . . . . . C . C C    
1144	. . . . . . . . . . . . . C . C C    
1145	. . . . . . . . . . . . . T . T T    
1146	. . . . . . . . . . . . . T . T T    
1147	. . . . . . . . . . . . . G . G G    
1148	. . . . . . . . . . . . . T . T T    
1149	. . . . . . . . . . . . . A . A A    
1150	. . . . . . . . . . . . . G . G G    
1151	. . . . . . . . . . . . . C . C C    
1152	. . . . . . . . . . . . . G . G G    
1153	. . . . . . . . . . . . . C . C C    
1154	. . . . . . . . . . . . . T . T T    
1155	. . . . . . . . . . . . . G . G G    
1156	. . . . . . . . . . . . . A . A A    
1157	. . . . . . . . . . . . . C . C C    
1158	. . . . . . . . . . . . . T . T T    
1159	. . . . . . . . . . . . . G . G G    
1160	. . . . . . . . . . . . . T . T T    
1161	. . . . . . . . . . . . . C . C C    
1162	. . . . . . . . . . . . . T . T T    
1163	. . . . . . . . . . . . . T . T T    
1164	. . . . . . . . . . . . . C . C C    
1165	. . . . . . . . . . . . . T . T T    
1166	. . . . . . . . . . . . . G . G G    
1167	. . . . . . . . . . . . . T . T T    
1168	. . . . . . . . . . . . . G . G G    
1169	. . . . . . . . . . . . . G . G G    
1170	. . . . . . . . . . . . . C . C C    
1171	. . . . . . . . . . . . . A . A A    
1172	. . . . . . . . . . . . . A . A A    
1173	. . . . . . . . . . . . . A . A A    
1174	. . . . . . . . . . . . . C . C C    
1175	. . . . . . . . . . . . . T . T T    
1176	. . . . . . . . . . . . . C . C C    
1177	. . . . . . . . . . . . . G . G G    
1178	. . . . . . . . . . . . . T . T T    
1179	. . . . . . . . . . . . . A . A A    
1180	. . . . . . . . . . . . . G . G G    
1181	. . . . . . . . . . . . . A . A A    
1182	. . . . . . . . . . . . . T . T T    
1183	. . . . . . . . . . . . . C . C C    
1184	. . . . . . . . . . . . . C . C C    
1185	. . . . . . . . . . . . . A . A A    
1186	. . . . . . . . . . . . . G . G G    
1187	. . . . . . . . . . . . . C . C C    
1188	. . . . . . . . . . . . . C . C C    
1189	. . . . . . . . . . . . . C . C C    
1190	. . . . . . . . . . . . . A . A A    
1191	. . . . . . . . . . . . . G . G G    
1192	. . . . . . . . . . . . . C . C C    
1193	. . . . . . . . . . . . . T . T T    
1194	. . . . . . . . . . . . . T . T T    
1195	. . . . . . . . . . . . . G . G G    
1196	. . . . . . . . . . . . . C . C C    
1197	. . . . . . . . . . . . . T . T T    
1198	. . . . . . . . . . . . . G . G G    
1199	. . . . . . . . . . . . . T . T T    
1200	. . . . . . . . . . . . . T . T T    
1201	. . . . . . . . . . . . . G . G G    
1202	. . . . . . . . . . . . . C . C C    
1203	. . . . . . . . . . . . . A . A A    
1204	. . . . . . . . . . . . . G . G G    
1205	. . . . . . . . . . . . . T . T T    
1206	. . . . . . . . . . . . . T . T T    
1207	. . . . . . . . . . . . . C . C C    
1208	. . . . . . . . . . . . . T . T T    
1209	. . . . . . . . . . . . . T . T T    
1210	. . . . . . . . . . . . . G . G G    
1211	. . . . . . . . . . . . . C . C C    
1212	. . . . . . . . . . . . . A . A A    
1213	. . . . . . . . . . . . . G . G G    
1214	. . . . . . . . . . . . . C . C C    
1215	. . . . . . . . . . . . . T . T T    
1216	. . . . . . . . . . . . . G . G G    
1217	. . . . . . . . . . . . . A . A A    
1218	. . . . . . . . . . . . . C . C C    
1219	. . . . . . . . . . . . . G . G G    
1220	. . . . . . . . . . . . . T . T T    
1221	. . . . . . . . . . . . . C . C C    
1222	. . . . . . . . . . . . . T . T T    
1223	. . . . . . . . . . . . . C . C C    
1224	. . . . . . . . . . . . . G . G G    
1225	. . . . . . . . . . . . . C . C C    
1226	. . . . . . . . . . . . . A . A A    
1227	. . . . . . . . . . . . . C . C C    
1228	. . . . . . . . . . . . . C . C C    
1229	. . . . . . . . . . . . . A . A A    
1230	. . . . . . . . . . . . . T . T T    
1231	. . . . . . . . . . . . . G . G G    
1232	. . . . . . . . . . . . . T . T T    
1233	. . . . . . . . . . . . . G . G G    
1234	. . . . . . . . . . . . . T . T T    
1235	. . . . . . . . . . . . . C . C C    
1236	. . . . . . . . . . . . . T . T T    
1237	. . . . . . . . . . . . . G . G G    
1238	. . . . . . . . . . . . . C . C C    
1239	. . . . . . . . . . . . . C . C C    
1240	. . . . . . . . . . . . . C . C C    
1241	. . . . . . . . . . . . . G . G G    
1242	. . . . . . . . . . . . . T . T T    
1243	. . . . . . . . . . . . . C . C C    
1244	. . . . . . . . . . . . . A . A A    
1245	. . . . . . . . . . . . . G . G G    
1246	. . . . . . . . . . . . . C . C C    
1247	. . . . . . . . . . . . . A . A A    
1248	. . . . . . . . . . . . . T . T T    
1249	. . . . . . . . . . . . . C . C C    
1250	. . . . . . . . . . . . . A . A A    
1251	. . . . . . . . . . . . . C . C C    
1252	. . . . . . . . . . . . . A . A A    
1253	. . . . . . . . . . . . . C . C C    
1254	. . . . . . . . . . . . . G . G G    
1255	. . . . . . . . . . . . . G . G G    
1256	. . . . . . . . . . . . . T . T T    
1257	. . . . . . . . . . . . . C C C C    
1258	. . . . . . . . . . . . . C C C C    
1259	. . . . . . . . . . . . . T T T T    
1260	. . . . . . . . . . . . . G G G G    
1261	. . . . . . . . . . . . . C C C C    
1262	. . . . . . . . . . . . . A A A A    
1263	. . . . . . . . . . . . . C C C C    
1264	. . . . . . . . . . . . . C C C C    
1265	. . . . . . . . . . . . . T T T T    
1266	. . . . . . . . . . . . . C C C C    
1267	. . . . . . . . . . . . . A A A A    
1268	. . . . . . . . . . . . . C C C C    
1269	. . . . . . . . . . . . . T T T T    
1270	. . . . . . . . . . . . . G G G G    
1271	. . . . . . . . . . . . . T T T T    
1272	. . . . . . . . . . . . . A A A A    
1273	. . . . . . . . . . . . . C C C C    
1274	. . . . . . . . . . . . . T T T T    
1275	. . . . . . . . . . . . . G G G G    
1276	. . . . . . . . . . . . . C C C C    
1277	. . . . . . . . . . . . . A A A A    
1278	. . . . . . . . . . . . . G G G G    
1279	. . . . . . . . . . . . . G G G G    
1280	. . . . . . . . . . . . . T T T T    
1281	. . . . . . . . . . . . . T T T T    
1282	. . . . . . . . . . . . . C C C C    
1283	. . . . . . . . . . . . . A A A A    
1284	. . . . . . . . . . . . . C C C C    
1285	. . . . . . . . . . . . . C C C C    
1286	. . . . . . . . . . . . . A A A A    
1287	. . . . . . . . . . . . . C C C C    
1288	. . . . . . . . . . . . . C C C C    
1289	. . . . . . . . . . . . . T T T T    
1290	. . . . . . . . . . . . . T T T T    
1291	. . . . . . . . . . . . . G G G G    
1292	. . . . . . . . . . . . . T T T T    
1293	. . . . . . . . . . . . . T T T T    
1294	. . . . . . . . . . . . . G G G G    
1295	. . . . . . . . . . . . . A A A A    
1296	. . . . . . . . . . . . . A A A A    
1297	. . . . . . . . . . . . . G G G G    
1298	. . . . . . . . . . . . . A A A A    
1299	. . . . . . . . . . . . . G G G G    
1300	. . . . . . . . . . . . . C G G G    
1301	. . . . . . . . . . . . . . A A A    
1302	. . . . . . . . . . . . . . A A A    
1303	. . . . . . . . . . . . . . G G G    
1304	. . . . . . . . . . . . . . G G G    
1305	. . . . . . . . . . . . . . C C C    
1306	. . . . . . . . . . . . . . T T T    
1307	. . . . . . . . . . . . . . C C C    
1308	. . . . . . . . . . . . . . G G G    
1309	. . . . . . . . . . . . . . G G G    
1310	. . . . . . . . . . . . . . C C C    
1311	. . . . . . . . . . . . . . C C C    
1312	. . . . . . . . . . . . . . C C C    
1313	. . . . . . . . . . . . . . G G G    
1314	. . . . . . . . . . . . . . T T T    
1315	. . . . . . . . . . . . . . G G G    
1316	. . . . . . . . . . . . . . G G G    
1317	. . . . . . . . . . . . . . C C C    
1318	. . . . . . . . . . . . . . G G G    
1319	. . . . . . . . . . . . . . C C C    
1320	. . . . . . . . . . . . . . C C C    
1321	. . . . . . . . . . . . . . A A A    
1322	. . . . . . . . . . . . . . G G G    
1323	. . . . . . . . . . . . . . T T T    
1324	. . . . . . . . . . . . . . G G G    
1325	. . . . . . . . . . . . . . A A A    
1326	. . . . . . . . . . . . . . A A A    
1327	. . . . . . . . . . . . . . G G G    
1328	. . . . . . . . . . . . . . C C C    
1329	. . . . . . . . . . . . . . G G G    
1330	. . . . . . . . . . . . . . C C C    
1331	. . . . . . . . . . . . . . G G G    
1332	. . . . . . . . . . . . . . T T T    
1333	. . . . . . . . . . . . . . G G G    
1334	. . . . . . . . . . . . . . G G G    
1335	. . . . . . . . . . . . . . A A A    
1336	. . . . . . . . . . . . . . G G G    
1337	. . . . . . . . . . . . . . A A A    
1338	. . . . . . . . . . . . . . T T T    
1339	. . . . . . . . . . . . . . G G G    
1340	. . . . . . . . . . . . . . A A A    
1341	. . . . . . . . . . . . . . G G G    
1342	. . . . . . . . . . . . . . C C C    
1343	. . . . . . . . . . . . . . T T T    
1344	. . . . . . . . . . . . . . C C C    
1345	. . . . . . . . . . . . . . A A A    
1346	. . . . . . . . . . . . . . G G G    
1347	. . . . . . . . . . . . . . A A A    
1348	. . . . . . . . . . . . . . G G G    
1349	. . . . . . . . . . . . . . C C C    
1350	. . . . . . . . . . . . . . G G G    
1351	. . . . . . . . . . . . . . G G G    
1352	. . . . . . . . . . . . . . T T T    
1353	. . . . . . . . . . . . . . T T T    
1354	. . . . . . . . . . . . . . G G G    
1355	. . . . . . . . . . . . . . G G G    
1356	. . . . . . . . . . . . . . T T T    
1357	. . . . . . . . . . . . . . C C C    
1358	. . . . . . . . . . . . . . A A A    
1359	. . . . . . . . . . . . . . G G G    
1360	. . . . . . . . . . . . . . G G G    
1361	. . . . . . . . . . . . . . A A A    
1362	. . . . . . . . . . . . . . T T T    
1363	. . . . . . . . . . . . . . G G G    
1364	. . . . . . . . . . . . . . G G G    
1365	. . . . . . . . . . . . . . T T T    
1366	. . . . . . . . . . . . . . G G G    
1367	. . . . . . . . . . . . . . T T T    
1368	. . . . . . . . . . . . . . C C C    
1369	. . . . . . . . . . . . . . G G G    
1370	. . . . . . . . . . . . . . C C C    
1371	. . . . . . . . . . . . . . A A A    
1372	. . . . . . . . . . . . . . G G G    
1373	. . . . . . . . . . . . . . T T T    
1374	. . . . . . . . . . . . . . T T T    
1375	. . . . . . . . . . . . . . G G G    
1376	. . . . . . . . . . . . . . G G G    
1377	. . . . . . . . . . . . . . C C C    
1378	. . . . . . . . . . . . . . G G G    
1379	. . . . . . . . . . . . . . C C C    
1380	. . . . . . . . . . . . . . A A A    
1381	. . . . . . . . . . . . . . G G G    
1382	. . . . . . . . . . . . . . G G G    
1383	. . . . . . . . . . . . . . A A A    
1384	. . . . . . . . . . . . . . G G G    
1385	. . . . . . . . . . . . . . A A A    
1386	. . . . . . . . . . . . . . A A A    
1387	. . . . . . . . . . . . . . G G G    
1388	. . . . . . . . . . . . . . A A A    
1389	. . . . . . . . . . . . . . G G G    
1390	. . . . . . . . . . . . . . G G G    
1391	. . . . . . . . . . . . . . C C C    
1392	. . . . . . . . . . . . . . G G G    
1393	. . . . . . . . . . . . . . G G G    
1394	. . . . . . . . . . . . . . G G G    
1395	. . . . . . . . . . . . . . T T T    
1396	. . . . . . . . . . . . . . C C C    
1397	. . . . . . . . . . . . . . C C C    
1398	. . . . . . . . . . . . . . C C C    
1399	. . . . . . . . . . . . . . G G G    
1400	. . . . . . . . . . . . . . C C C    
1401	. . . . . . . . . . . . . . C C C    
1402	. . . . . . . . . . . . . . G G G    
1403	. . . . . . . . . . . . . . A A A    
1404	. . . . . . . . . . . . . . T T T    
1405	. . . . . . . . . . . . . . G G G    
1406	. . . . . . . . . . . . . . T T T    
1407	. . . . . . . . . . . . . . G G G    
1408	. . . . . . . . . . . . . . G G G    
1409	. . . . . . . . . . . . . . T T T    
1410	. . . . . . . . . . . . . . C C C    
1411	. . . . . . . . . . . . . . C C C    
1412	. . . . . . . . . . . . . . A A A    
1413	. . . . . . . . . . . . . . G G G    
1414	. . . . . . . . . . . . . . G G G    
1415	. . . . . . . . . . . . . . A A A    
1416	. . . . . . . . . . . . . . A A A    
1417	. . . . . . . . . . . . . . G G G    
1418	. . . . . . . . . . . . . . A A A    
1419	. . . . . . . . . . . . . . T T T    
1420	. . . . . . . . . . . . . . C C C    
1421	. . . . . . . . . . . . . . C C C    
1422	. . . . . . . . . . . . . . G G G    
1423	. . . . . . . . . . . . . . G G G    
1424	. . . . . . . . . . . . . . C C C    
1425	. . . . . . . . . . . . . . C C C    
1426	. . . . . . . . . . . . . . C C C    
1427	. . . . . . . . . . . . . . A A A    
1428	. . . . . . . . . . . . . . T T T    
1429	. . . . . . . . . . . . . . A A A    
1430	. . . . . . . . . . . . . . G G G    
1431	. . . . . . . . . . . . . . T T T    
1432	. . . . . . . . . . . . . . G G G    
1433	. . . . . . . . . . . . . . C C C    
1434	. . . . . . . . . . . . . . T T T    
1435	. . . . . . . . . . . . . . G G G    
1436	. . . . . . . . . . . . . . T T T    
1437	. . . . . . . . . . . . . . T T T    
1438	. . . . . . . . . . . . . . G G G    
1439	. . . . . . . . . . . . . . T T T    
1440	. . . . . . . . . . . . . . C C C    
1441	. . . . . . . . . . . . . . A A A    
1442	. . . . . . . . . . . . . . G G G    
1443	. . . . . . . . . . . . . . C C C    
1444	. . . . . . . . . . . . . . A A A    
1445	. . . . . . . . . . . . . . G G G    
1446	. . . . . . . . . . . . . . A A A    
1447	. . . . . . . . . . . . . . G G G    
1448	. . . . . . . . . . . . . . A A A    
1449	. . . . . . . . . . . . . . C C C    
1450	. . . . . . . . . . . . . . C C C    
1451	. . . . . . . . . . . . . . G G G    
1452	. . . . . . . . . . . . . . A A A    
1453	. . . . . . . . . . . . . . C C C    
1454	. . . . . . . . . . . . . . C C C    
1455	. . . . . . . . . . . . . . T T T    
1456	. . . . . . . . . . . . . . T T T    
1457	. . . . . . . . . . . . . . G G G    
1458	. . . . . . . . . . . . . . A A A    
1459	. . . . . . . . . . . . . . A A A    
1460	. . . . . . . . . . . . . . T T T    
1461	. . . . . . . . . . . . . . C C C    
1462	. . . . . . . . . . . . . . T T T    
1463	. . . . . . . . . . . . . . G G G    
1464	. . . . . . . . . . . . . . T T T    
1465	. . . . . . . . . . . . . . A A A    
1466	. . . . . . . . . . . . . . A A A    
1467	. . . . . . . . . . . . . . T T T    
1468	. . . . . . . . . . . . . . T T T    
1469	. . . . . . . . . . . . . . T T T    
1470	. . . . . . . . . . . . . . T T T    
1471	. . . . . . . . . . . . . . C C C    
1472	. . . . . . . . . . . . . . C C C    
1473	. . . . . . . . . . . . . . C C C    
1474	. . . . . . . . . . . . . . G G G    
1475	. . . . . . . . . . . . . . A A A    
1476	. . . . . . . . . . . . . . G G G    
1477	. . . . . . . . . . . . . . T T T    
1478	. . . . . . . . . . . . . . T T T    
1479	. . . . . . . . . . . . . . A A A    
1480	. . . . . . . . . . . . . . T T T    
1481	. . . . . . . . . . . . . . G G G    
1482	. . . . . . . . . . . . . . T T T    
1483	. . . . . . . . . . . . . . C C C    
1484	. . . . . . . . . . . . . . G G G    
1485	. . . . . . . . . . . . . . A A A    
1486	. . . . . . . . . . . . . . C C C    
1487	. . . . . . . . . . . . . . A A A    
1488	. . . . . . . . . . . . . . C C C    
1489	. . . . . . . . . . . . . . G G G    
1490	. . . . . . . . . . . . . . A A A    
1491	. . . . . . . . . . . . . . A A A    
1492	. . . . . . . . . . . . . . T T T    
1493	. . . . . . . . . . . . . . G G G    
1494	. . . . . . . . . . . . . . T T T    
1495	. . . . . . . . . . . . . . T T T    
1496	. . . . . . . . . . . . . . A A A    
1497	. . . . . . . . . . . . . . A A A    
1498	. . . . . . . . . . . . . . T T T    
1499	. . . . . . . . . . . . . . A A A    
1500	. . . . . . . . . . . . . . A A A    
1501	. . . . . . . . . . . . . . C C C    
1502	. . . . . . . . . . . . . . C C C    
1503	. . . . . . . . . . . . . . T T T    
1504	. . . . . . . . . . . . . . G G G    
1505	. . . . . . . . . . . . . . T T T    
1506	. . . . . . . . . . . . . . T T T    
1507	. . . . . . . . . . . . . . T T T    
1508	. . . . . . . . . . . . . . T T T    
1509	. . . . . . . . . . . . . . C C C    
1510	. . . . . . . . . . . . . . T T T    
1511	. . . . . . . . . . . . . . T T T    
1512	. . . . . . . . . . . . . . G G G    
1513	. . . . . . . . . . . . . . A A A    
1514	. . . . . . . . . . . . . . C C C    
1515	. . . . . . . . . . . . . . C C C    
1516	. . . . . . . . . . . . . . A A A    
1517	. . . . . . . . . . . . . . C C C    
1518	. . . . . . . . . . . . . . T T T    
1519	. . . . . . . . . . . . . . T T T    
1520	. . . . . . . . . . . . . . C C C    
1521	. . . . . . . . . . . . . . T T T    
1522	. . . . . . . . . . . . . . A A A    
1523	. . . . . . . . . . . . . . C C C    
1524	. . . . . . . . . . . . . . A A A    
1525	. . . . . . . . . . . . . . G G G    
1526	. . . . . . . . . . . . . . T T T    
1527	. . . . . . . . . . . . . . C C C    
1528	. . . . . . . . . . . . . . T T T    
1529	. . . . . . . . . . . . . . T T T    
1530	. . . . . . . . . . . . . . T T T    
1531	. . . . . . . . . . . . . . A A A    
1532	. . . . . . . . . . . . . . T T T    
1533	. . . . . . . . . . . . . . C C C    
1534	. . . . . . . . . . . . . . A A A    
1535	. . . . . . . . . . . . . . A A A    
1536	. . . . . . . . . . . . . . C C C    
1537	. . . . . . . . . . . . . . C C C    
1538	. . . . . . . . . . . . . . T T T    
1539	. . . . . . . . . . . . . . C C C    
1540	. . . . . . . . . . . . . . G G G    
1541	. . . . . . . . . . . . . . A A A    
1542	. . . . . . . . . . . . . . A A A    
1543	. . . . . . . . . . . . . . T T T    
1544	. . . . . . . . . . . . . . C C C    
1545	. . . . . . . . . . . . . . C C C    
1546	. . . . . . . . . . . . . . A A A    
1547	. . . . . . . . . . . . . . A A A    
1548	. . . . . . . . . . . . . . T T T    
1549	. . . . . . . . . . . . . . T T T    
1550	. . . . . . . . . . . . . . T T T    
1551	. . . . . . . . . . . . . . T T T    
1552	. . . . . . . . . . . . . . G G G    
1553	. . . . . . . . . . . . . . T T T    
1554	. . . . . . . . . . . . . . T T T    
1555	. . . . . . . . . . . . . . G G G    
1556	. . . . . . . . . . . . . . T T T    
1557	. . . . . . . . . . . . . . T T T    
1558	. . . . . . . . . . . . . . G G G    
1559	. . . . . . . . . . . . . . T T T    
1560	. . . . . . . . . . . . . . A A A    
1561	. . . . . . . . . . . . . . T T T    
1562	. . . . . . . . . . . . . . T T T    
1563	. . . . . . . . . . . . . . T T T    
1564	. . . . . . . . . . . . . . T T T    
1565	. . . . . . . . . . . . . . C C C    
1566	. . . . . . . . . . . . . . A A A    
1567	. . . . . . . . . . . . . . C C C    
1568	. . . . . . . . . . . . . . C C C    
1569	. . . . . . . . . . . . . . C C C    
1570	. . . . . . . . . . . . . . T T T    
1571	. . . . . . . . . . . . . . T T T    
1572	. . . . . . . . . . . . . . C C C    
1573	. . . . . . . . . . . . . . T T T    
1574	. . . . . . . . . . . . . . T T T    
1575	. . . . . . . . . . . . . . C C C    
1576	. . . . . . . . . . . . . . G G G    
1577	. . . . . . . . . . . . . . T T T    
1578	. . . . . . . . . . . . . . C C C    
1579	. . . . . . . . . . . . . . C C C    
1580	. . . . . . . . . . . . . . T T T    
1581	. . . . . . . . . . . . . . C C C    
1582	. . . . . . . . . . . . . . G G G    
1583	. . . . . . . . . . . . . . A A A    
1584	. . . . . . . . . . . . . . T T T    
1585	. . . . . . . . . . . . . . T T T    
1586	. . . . . . . . . . . . . . C C C    
1587	. . . . . . . . . . . . . . T T T    
1588	. . . . . . . . . . . . . . G G G    
1589	. . . . . . . . . . . . . . T T T    
1590	. . . . . . . . . . . . . . A A A    
1591	. . . . . . . . . . . . . . G G G    
1592	. . . . . . . . . . . . . . C C C    
1593	. . . . . . . . . . . . . . T T T    
1594	. . . . . . . . . . . . . . A A A    
1595	. . . . . . . . . . . . . . G G G    
1596	. . . . . . . . . . . . . . A A A    
1597	. . . . . . . . . . . . . . T T T    
1598	. . . . . . . . . . . . . . G G G    
1599	. . . . . . . . . . . . . . T T T    
1600	. . . . . . . . . . . . . . G G G    
1601	. . . . . . . . . . . . . . G G G    
1602	. . . . . . . . . . . . . . C C C    
1603	. . . . . . . . . . . . . . G G G    
1604	. . . . . . . . . . . . . . T T T    
1605	. . . . . . . . . . . . . . C C C    
1606	. . . . . . . . . . . . . . A A A    
1607	. . . . . . . . . . . . . . G G G    
1608	. . . . . . . . . . . . . . C C C    
1609	. . . . . . . . . . . . . . T T T    
1610	. . . . . . . . . . . . . . T T T    
1611	. . . . . . . . . . . . . . T T T    
1612	. . . . . . . . . . . . . . G G G    
1613	. . . . . . . . . . . . . . T T T    
1614	. . . . . . . . . . . . . . T T T    
1615	. . . . . . . . . . . . . . G G G    
1616	. . . . . . . . . . . . . . T T T    
1617	. . . . . . . . . . . . . . T T T    
1618	. . . . . . . . . . . . . . C C C    
1619	. . . . . . . . . . . . . . T T T    
1620	. . . . . . . . . . . . . . C C C    
1621	. . . . . . . . . . . . . . G T T    
1622	. . . . . . . . . . . . . . . C C    
1623	. . . . . . . . . . . . . . . A A    
1624	. . . . . . . . . . . . . . . C C    
1625	. . . . . . . . . . . . . . . A A    
1626	. . . . . . . . . . . . . . . G G    
1627	. . . . . . . . . . . . . . . C C    
1628	. . . . . . . . . . . . . . . T T    
1629	. . . . . . . . . . . . . . . G G    
1630	. . . . . . . . . . . . . . . T T    
1631	. . . . . . . . . . . . . . . T T
```
